# Neuroergonomics Applications of Electroencephalography in Physical Activities: A Systematic Review

**DOI:** 10.3389/fnhum.2019.00182

**Published:** 2019-06-04

**Authors:** Mahjabeen Rahman, Waldemar Karwowski, Magdalena Fafrowicz, Peter A. Hancock

**Affiliations:** ^1^Computational Neuroergonomics Laboratory, Department of Industrial Engineering and Management Systems, University of Central Florida, Orlando, FL, United States; ^2^Department of Cognitive Neuroscience and Neuroergonomics, Institute of Applied Psychology, Jagiellonian University in Krakow, Krakow, Poland; ^3^Department of Psychology, University of Central Florida, Orlando, FL, United States

**Keywords:** EEG, neuroergonomics, ergonomics, physical exercise, physical activity, physical exertion, brain, human factors

## Abstract

Recent years have seen increased interest in neuroergonomics, which investigates the brain activities of people engaged in diverse physical and cognitive activities at work and in everyday life. The present work extends upon prior assessments of the state of this art. However, here we narrow our focus specifically to studies that use electroencephalography (EEG) to measure brain activity, correlates, and effects during physical activity. The review uses systematically selected, openly published works derived from a guided search through peer-reviewed journals and conference proceedings. Identified studies were then categorized by the type of physical activity and evaluated considering methodological and chronological aspects via statistical and content-based analyses. From the identified works (*n* = 110), a specific number (*n* = 38) focused on less mobile muscular activities, while an additional group (*n* = 22) featured both physical and cognitive tasks. The remainder (*n* = 50) investigated various physical exercises and sporting activities and thus were here identified as a miscellaneous grouping. Most of the physical activities were isometric exertions, moving parts of upper and lower limbs, or walking and cycling. These primary categories were sub-categorized based on movement patterns, the use of the event-related potentials (ERP) technique, the use of recording methods along with EEG and considering mental effects. Further information on subjects' gender, EEG recording devices, data processing, and artifact correction methods and citations was extracted. Due to the heterogeneous nature of the findings from various studies, statistical analyses were not performed. These were thus included in a descriptive fashion. Finally, contemporary research gaps were pointed out, and future research prospects to address those gaps were discussed.

## Introduction

Neuroergonomics is the study of the brain's structure and function, cognition, and behavioral output during work or other real-world activities (Parasuraman and Rizzo, 2008). Neuroergonomics is an emerging field within both human factors and ergonomics (HFE) as well as the wider neurosciences. HFE strives for human-compatible systems by applying human-centered principles (Karwowski, 2005). Beyond its conventional applications, HFE aims to ensure efficient systems design in numerous special cases, e.g., for those with functional disabilities, aging populations, in military contexts, and for student and trainee populations (see Marek et al., 2010). Neuroergonomics plays a unique role in each of these fields, which each require their own specific assessment methods. However, in general, neuroimaging methods are currently preferred over other neuroergonomics research methods, such as subjective workload (cf., Parasuraman and Rizzo, 2008; Hancock and Matthews, 2019). The present review takes advantage of a prior but more general assessment by Mehta and Parasuraman (2013) published more than 5 years ago.

Neuroimaging methods are used to observe and analyze brain activity. The human brain is a uniquely sophisticated organ that renders humans the most intelligent known species. The brain contains approximately 86 billion neurons (Azevedo et al., 2009). These neurons continuously make connections with one another in the form of electrical signals (Stern et al., 2001). Neuronal firings generate different types of wave patterns, which emanate from differing brain regions. These include delta (<4 Hz), theta (4–7 Hz), alpha (7–12 Hz), beta (12–30 Hz), gamma (30–50 Hz), and mu (9–11 Hz) waveforms (Niedermeyer and da Silva, 2005). Alpha waves, originally designated as the “Berger rhythm”, are taken to represent a relaxed state. Alpha waves are one of the most widely studied frequency patterns and exhibit a general inverse relation with cortical activation (Teplan, 2002). One variation of alpha is designated the mu rhythm, which originates from the motor cortex and plays a role in voluntary movement (Niedermeyer and da Silva, 2005).

The voluntary control of muscle movements and motor activities is a crucial function of the brain (Hancock and Newell, 1985; Karwowski et al., 2003). Research in physical neuroergonomics emphasizes the cerebral cortex, which is involved in controlling muscle activation and smoothing high-speed motor control processes (Karwowski et al., 2003). Perhaps the most commonly used neuroimaging technique in neuroergonomics is electroencephalography (EEG) (Parasuraman and Wilson, 2008). EEG is a noninvasive method whereby electrodes are placed on the scalp to measure spontaneous electrical activities of the brain (Niedermeyer and da Silva, 2005). Parasuraman and Rizzo (2008) suggested that brain monitoring for ergonomic studies should be robust, sensitive, unobtrusive, and economical, and should have a high temporal resolution. The EEG technique satisfies all of these criteria as well as holding the promise for effective capacities in more challenging real-world conditions (Sawyer et al., 2017).

EEG studies, with a focus on physical activity, are widespread across reports from differing fields. There have yet to be any published reviews in neuroergonomics that have focused specifically on EEG. Given the significance of EEG and the contemporary body of published work, it is now justifiable to conduct such a search and assessment. There is a necessity to investigate current literature and to determine whether these works can be classified and synthesized accordingly. Furthermore, for EEG studies, methodological issues, e.g., EEG device, data processing, and data cleaning, are significant aspects to consider. The information from present works can be beneficial to both current and future investigations. In addition, it is essential to determine from published studies whether there is a research gap from the perspective of neuroergonomics, i.e., using ecologically valid settings. Our primary goal is to categorize and consolidate the published experimental EEG studies with participants engaged in moving one or more parts of their body. The specific research issues addressed in the resultant review therefore are:

- What are the published EEG studies on physical neuroergonomics and how can these be categorized?
- What are the current research gaps and what are the future research prospects in applying EEG for brain monitoring purposes when there is physical activity?

## Methods

### Protocol

The present literature review was conducted in accordance with the Preferred Reporting Items for Systematic Reviews and Meta-analyses (PRISMA) guidelines (Moher et al., 2009). The applied protocol was developed, predicated upon the above-identified specific research questions and using the specified search strategy described below.

### Search Strategy

The literature search was performed using several databases explored through Boolean operators and specified search terms, i.e., (“EEG” OR “Electroencephalogram”) AND (“physical activity” OR “physical exertion” OR “physical movement” OR “exercise”). For some databases, e.g., Compendex, a secondary search was then conducted by controlling for these keywords on the basis of the prior results distinguished. Similarly, other databases were searched including Web of Science, Engineering Village Compendex, EBSCO Host, ProQuest, PsychINFO, and Google Scholar. In addition to these databases, papers from the common collection of fellow researchers were screened for eligibility. Two researchers (MR and WK) screened all of the papers and resolved discrepancies through regular discussion. Thus, we ensured minimized bias and that the most relevant works were identified in every search. Concordance among results for different databases also indicated that the intended articles were retrieved.

The following set criteria were used to screen identified sources.

- Papers were presented in English only.
- They appeared in peer-reviewed journal or conference publications.
- They focused on a non-clinical population, i.e., reported experimental work conducted on healthy human subjects of any gender.
- They were published until 20th December, 2018 (last search date).

**Figure 1:**
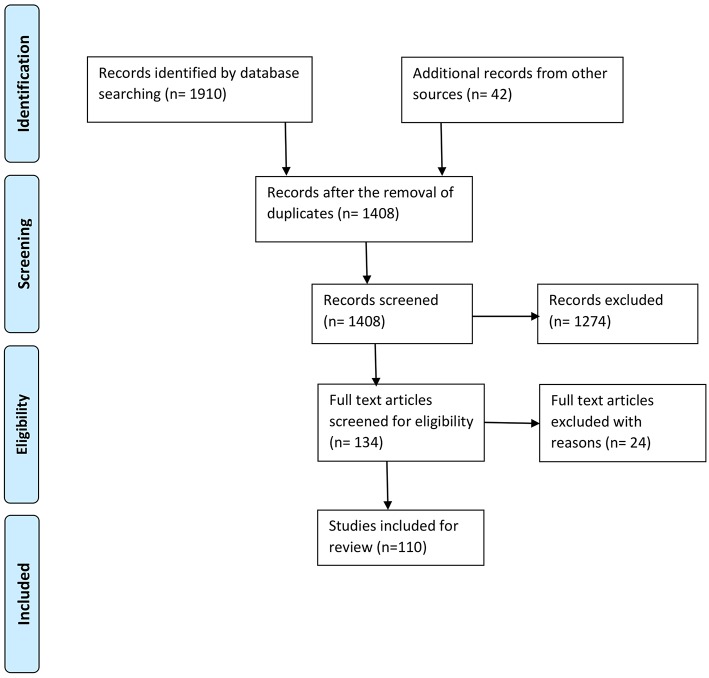
Process of selection of the studies according to PRISMA (Moher et al., 2009).

Excluded works were those which were not based on an experimental analyses, did not present original research, did not relate to physical movement(s) or activities, focused on specific neural disorders or brain diseases, or included infants or children as participants. Books, book chapters, or review papers not related to the research questions, as well as opinions, viewpoints, blog posts, letters, and editorials, were excluded. Gray literature and reports were also precluded from further evaluation. Our overall search process and the associated quantitative identifications are shown in Figure 1.

### Data Extraction and Synthesis

**Figure 2:**
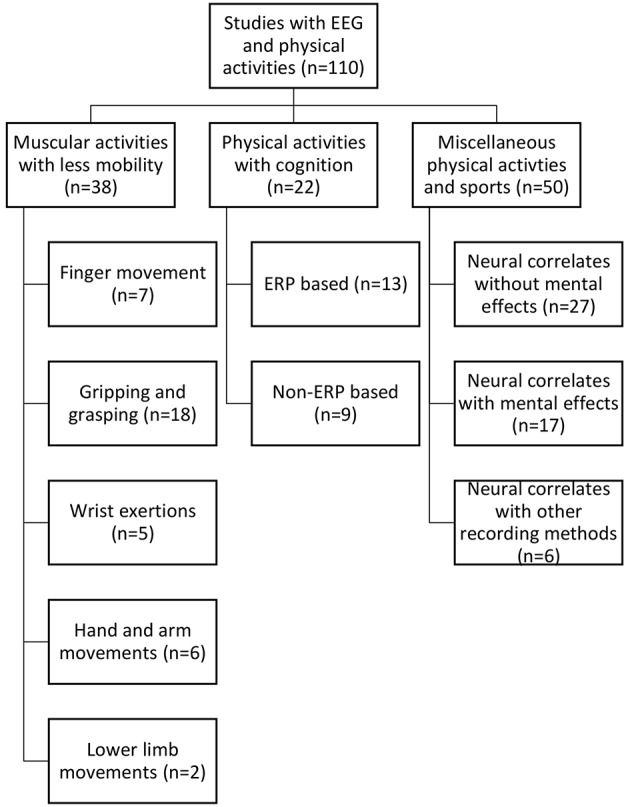
Taxonomy of the reviewed studies.

Having followed the identified selection process detailed above, the selected studies were organized primarily into three categories based on the type of activity, i.e., muscular activity with less mobility, physical activity with cognition and miscellaneous activities. For each study, a number of informational elements were extracted: published year, participant's gender, physical activity performed, data processing and analysis method, number of citations according to Google Scholar at the time the search was conducted, number of electrodes used by the EEG recording device and artifact correction method. The detailed statistical results from each study were not extracted due to their heterogeneous nature. All information was stored and organized in multiple spreadsheets. MR managed the data in spreadsheets. Each of the primary categories, as mentioned earlier, were further stratified based on a certain common feature. These common features were identified by observing the activity patterns, use of event-related potentials (ERP), use of mental effects, data collection methods, etc. Physical activities with less mobility were categorized into five divisions based on the type of movement pattern: (i) finger movement, (ii) gripping/grasping, (iii) hand and arm movement, (iv) lower limb movement, and (v) wrist exertions. Physical activities with cognition were divided into two categories, i.e., those that were ERP-based and non-ERP-based. Finally, a miscellaneous category was organized into three segments: neural correlates without mental effects, neural correlates with mental effects and neural correlates with recording methods along with EEG. An illustration of this taxonomy is provided in Figure 2.

## Results

**Figure 3:**
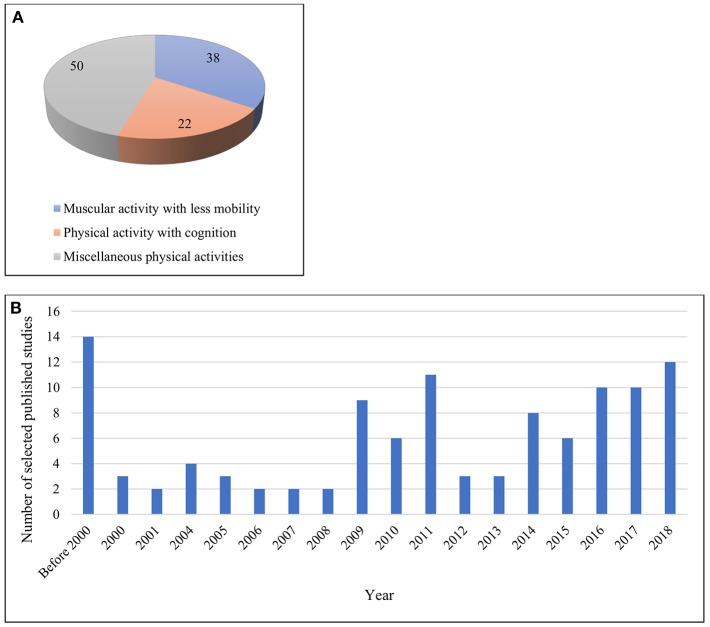
**(A)** Works across different categories **(B)** Works published over year.

A total of 110 articles were retrieved for final inclusion in the review. As specified earlier, these identified papers were divided into three broad categories. Approximately 50% of the studies included both sexes as participants. Of the total papers, (*n* = 38) were on muscular activity with less mobility. Of these, (*n* = 12) were focused on isometric/isotonic exertions. In addition, (*n* = 22) studies involved both physical and cognitive activities in combination. The remaining works (*n* = 50) were placed in the miscellaneous category, including a wide variety of differing physical pursuits and sporting activities, e.g., cycling, walking, physical exercise, load lifting, yoga, shooting, and others. However, of these latter (*n* = 50) papers, (n+30) included walking- or cycling-based activities. Figures 3A,B illustrate these studies according to their categories and over time, respectively.

**Figure 4:**
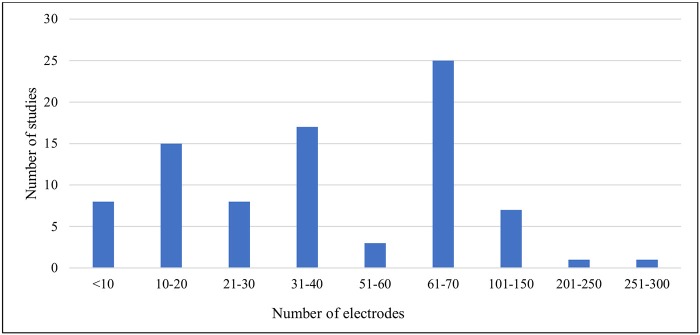
Number of recording electrodes versus number of studies included.

The demographic distribution of the studies included both healthy male and female participants. Of these, (*n* = 27) were with males only, (*n* = 3) with females only and (*n* = 50) with both genders. In addition, a further critical aspect of any EEG study is the recording electrodes used to collect data of sufficient quality for necessary interpretation (Teplan, 2002). Luck (2014) did not recommend recording with more than 30–40 electrodes because obtaining unequivocal data becomes difficult in such cases. It has also been noted that there are issues with using more than a full suite of 64 electrodes. The number of electrodes varied in the works we reviewed, and Figure 4 illustrates that most of the studies fulfilled the recommended conditions. However, (*n* = 8) studies used devices with 70+ electrodes.

After EEG data collection, one of the most crucial steps concerns the correction or rejection of waveform artifacts. Of note, it is always better not to rely heavily on artifact correction by naturally seeking to collect data that are as clean as possible (Luck, 2014). Studies involving physical movement are bound to generate artifacts due to movement of the limbs, muscles, skin, etc. Therefore, the artifact correction methods used in the studies with physical activities were herein specifically identified and examined. A majority of the studies (*n* = 31) used manual/visual correction methods, along with limited use of formal tools and algorithms (see Supplementary Tables). Finally, two types of summaries were developed. The first is a concise summary of the methodological aspects, documented in Table 1. Tables 2–4, include major findings from the studies. Furthermore, Supplementary Tables 1–3 consist of other significant information extracted from the papers corresponding to the three primary categories.

**Table 1:** A comprehensive summary of the findings from the review.

**Physical activity-based EEG studies**			
**Category of activity**	**Number of recording electrodes used**	**Data collection and processing method**	**Artifact correction method**
• Muscular activity with less mobility • Physical activity with cognition • Miscellaneous physical activities	2 to 264	• EEG-EMG coherence • n:m coherence • CMC • MRCP • MRP • ERP • ERD/ERS • ERSP • sLORETA • LORETA • PAF • PSD • Time-frequency analysis • Time-frequency decomposition • SDTF • CNV • FAA • EEG asymmetry • Microstate analysis • Nonlinear analysis • Fuzzy Entropy • Brain functional network (BFN) • Hierarchical linear model (HLM) • Dynamic casual modeling (DCM) • Feature extraction • Source reconstruction • Alpha-beta ratio • Band amplitudes spectrum • Biphasic paroxysmal sharp waves (BPSW)	• Visual/manual inspection • Independent component analysis (ICA) • AMICA • DIPFIT • Channel/epochs/trials rejection • Bad channel interpolation • EOG correction • EMG-based correction • Spatial filter • Source minimization • Trend correction • Topographic interpolation • Semi-automatic artifact rejection

**Table 2:** Major findings from studies under muscular activity with less mobility.

	**Category of muscular activity with less mobility**	**Major findings**
1	Finger movement	• Changes in signals due to changes in task complexity • Beta synchronization when low intensity flexion, extension • EEG-EMG coherence • Synchronization likelihood highest during rest • Mirror neurons activity reflecting observation and extension • Beta and gamma CMC correlations with force levels • Increases in MRCP and perceived exertion with trajectory of force
2	Gripping and grasping	• Time-dependent source strength function not correlated with force levels • Weak mu and beta ERDs during “hold” • Changes in cortical activation along with intensity of muscle activity • Rapid and distinct changes in cortical patterns during preparation, execution and feedback • Consistent beta modulation for different forces • No change in EEG during movement preparation despite high fatigue • Alternating motor centers retains optimal output during fatigue • Lower PAF indicates fatigue • More power in parietal and occipital areas for grip-based force modulation is needed for older ones • BOLD correlates with alpha and beta • Fractal dimensions positively correlate with grip force • L1 correlates with force and fatigue
3	Wrist exertions	• Alpha correlates with voluntary movement • Beta and gamma index in central electrode detect motor circuit activation • Lower beta power and peak beta CMC during flexion, opposite in extension • Stronger coherence between primary sensorimotor and motor association cortices • 46% relation between joint manipulation and cortical potentials
4	Hand and arm movements	• Increase in PAF when eyes closed • Force-dependent differences in neural signatures during preparation and initiation • Delta, theta and gamma are most sensitive during straight and spiral trajectory movements • Alpha power is less when there is load compared to no-load • Alpha is high in fatigue
5	Lower limb movements	• Beta and gamma coherence during static and dynamic force outputs respectively • CMC correlates positively with beta EMG and force fluctuation capacity

**Table 3:** Major findings from studies under physical activity with cognition.

	**Category of physical activity with cognition**	**Major findings**
1	ERP-based	• ERP signals increase in PMC for MP and physical execution • CNV signals during MVC and MP • P300 increases with physical activity • No alpha when there's no cognitive task • Presence of mu-ERD in 60% of walking steps • P1 increases in parieto-occipital areas during low-intensity exercise • Peak latency of P3a decreases for high and low intensity exercises • No significant difference between P300 and N400 across fitness levels • Lower ERN amplitudes for fitter subjects • Theta and alpha increase when there is cognition; also P3b increases • Posterior P3 decreases during physical and cognitive tasks • Fronto-central N2 and theta increase during Sudoku solving and physical task • Alpha activity and error rate increase in monotonous cognitive task
2	Non-ERP-based	• Bursts of EEG signals in motor and frontal cortex when pressing a button • Sensorimotor areas are more active during MI or MP • PFC is most active during CAT • PMC and SMA are most active during execution and imagery respectively • PMC-DLPFC and PMC-SMA couplings increase for correct and incorrect imagery respectively • Alpha and theta increase in posterior and fronto-central areas during a Qigong exercise • Increase in alpha and beta power when cognitively aroused • Theta power increases when there is mental fatigue • Beta CMC decreases after leg resistance exercise • Visual cortex is more sensitive during active movement • Beta amplitudes decrease to improve attention and task performance after neurofeedback • Evolution of theta, alpha and beta frequencies during traction exercise with visual feedback • Active right and central parietal areas after an auditory distractor

**Table 4:** Major findings from studies under miscellaneous physical activities.

	**Category of miscellaneous activities**	**Major findings**
1	Neural correlates without mental effects	• Alpha increases initially and then decreases when maximum running intensity is achieved • Beta amplitudes in occipital midline are inversely correlated with participants' physical condition • Alpha increases after exercise in parietal and occipital areas • Brain more active between exercise and no-exercise conditions • Increase of alpha immediately before physical task • Alpha increases, theta increases and beta decreases during lower brain activation • Decreased frequencies during repetitive knee-bending • Sharp patterns and reproducible waves during hatha-yoga • Increase in all major bands after gradually intensifying cycling • Increase in theta, alpha and beta amplitudes after moderate intensity running • Alpha decreases when there is high aerobic demand • More changes in alpha-beta ratio values in hot environment • Changes in gamma power between steady walking steps, intra-stride gamma frequency increases, alpha and beta frequency decrease in leg-motor areas • Changes in MRCP for squats of different intensity • Beta and gamma amplitudes increase during weight training • Mu power decreases during lifting prior to onset • Mean alpha power correlates with load levels of light assembly task • Greater activation in parietal-frontal areas in case of unfamiliar tool handling • Reduction in frontal midline theta and upper alpha power during the onset of golf putting • EEG signatures in left hemisphere correlates with shooting performance in archery
2	Neural correlates with mental effects	• More active left hemisphere when exercising with positive music, larger alpha in central areas when exercising with audiobook • Music improves motor control • In hot environment, perceived exertion correlates more with EEG than EMG • Hyperthermia due to cycling slows down EEG signals and increases RPE • Alpha and beta decrease in frontal and central areas due to exercise in hot environment • Reduced alpha when there is hypoxia, beta is higher than hot environment • Lagged phase synchronization increase at the end of cycling, coherence is less at the end of lifting • Fatigue and cortical amplitudes are proportional in resistance squats • Positive correlation between FAA and mood disturbance in older people, negative correlation for younger ones • Robust change in FAA after intense exercise are higher than at rest in younger females • Cortical activity correlates with exercise preference and task duration • Correlations among perceptions, psychological stress, alpha and beta • Resting FAA predicts affective state at a below threshold intensity level • No change in FAA for self-selected exercise, but better affective response • Decrease in frontal-midline theta and increase in temporal-occipital alpha in pre-shooting phase • Higher theta, lower left-central alpha and higher left-temporal alpha when there is higher shooting accuracy
3	Neural correlates with other recording methods	• EMG cyclic power modulation (24-40 Hz) for walking and cycling • Stronger and sustained cortical activation including decreased beta power in cycling, decreased alpha power in walking • EEG precedes EMG in low gamma band between swing and stance during gait • Beta suppression increases with force levels for strength training • Less activation of antagonist muscles in EMG for strength training than endurance training. • Reduced beta ERD in left sensorimotor areas, no correlation between CMC and motor skill retention • Motor neurons activate faster in regular exercisers • FuzzyEn and AMHRR are correlated in alpha, beta and theta bands

## Discussion

The present section provides a description of the major findings from the works under the three categories identified above.

### Muscular Activity With Less Mobility

**Figure 5:**
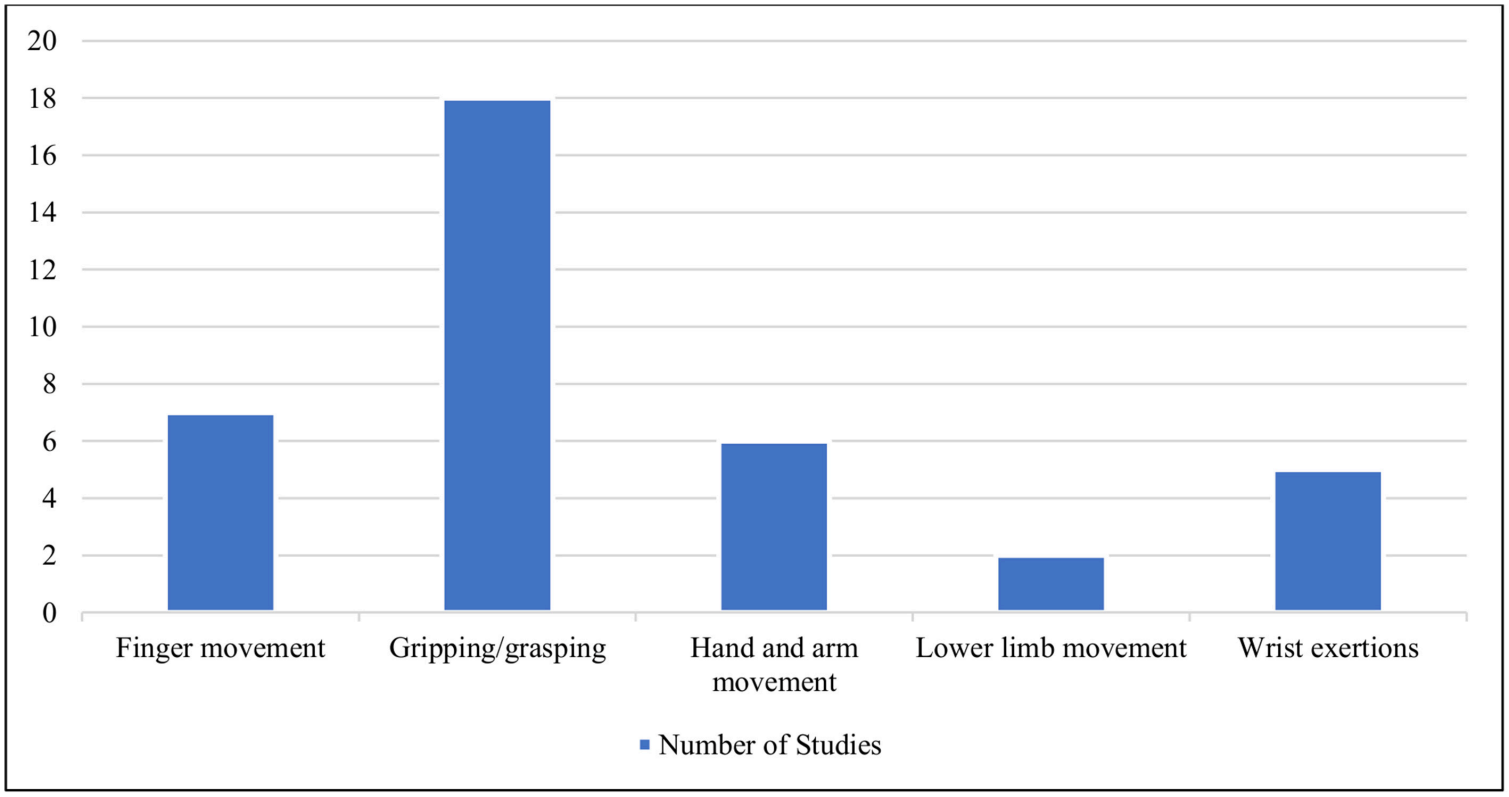
Different types of movement patterns under the category of physical activity with less mobility.

The works that focused on physical activity with little or no actual movement involved were designated in this category. Some of these tasks include gripping and grasping motions, moving the fingers, and moving the muscles of the wrist, arm, ankle, knee, and others. The works (*n* = 38) found with this type of activity were categorized according to the type of movement pattern. Many of these articles were focused on isometric/isotonic exertions. These studies are summarized in Supplementary Table 1, Table 2 and visualized in Figure 5.

#### Finger Movement

Among the earlier works, Breitling et al. (1986) demonstrated that increasing the complexity of finger movement tasks resulted in changes in the signal amplitudes in different brain regions, i.e., right frontal, prefrontal, posterior parietal and left temporal. Investigators have also used both EEG and electromyography (EMG) to study the effects of low-intensity activities on the brain. For voluntary phasic activity, such as flexion and extension of the index finger, beta synchronization of EEG and EMG was observed for the first time in the study by Feige et al. (2000). Through synthetic and experimental analyses, later studies demonstrated that a small number of motor neurons are involved in conveying cortical information. Moreover, EEG-EMG coherence was generated for these types of finger movements (Negro and Farina, 2011). Synchronization likelihood (SL) was largest during rest, thus indicating stable information processing in the brain. Calmels et al. (2006) suggested that mirror neurons may be responsible for these synchronization patterns during observation and then execution of the finger movement task.

For different types of force levels in an isometric finger movement, a broad band of cortico-muscular coherence (CMC), including beta and gamma activity, has been observed (Chakarov et al., 2009). These were represented in the form of positive correlations with the force level. However, further findings showed that the peak beta band (22 Hz) and gamma range CMCs are not significantly modulated by force levels. In addition to EEG-EMG coherence, other parameters such as movement-related cortical potential (MRCP) were employed. While performing an isometric index finger task, the perceived exertion and MRCP amplitudes increased with increases in the rate and level of force, respectively (Slobounov et al., 2004). Interestingly, for the lowest level of exerted force by the index finger only, the trajectory of force and EEG time series were highly correlated. For the other fingers, it appeared to be little in terms of significant differences in EEG signals (Slobounov et al., 2002).

#### Gripping and Grasping

The time-dependent relationship between EEG source strength and force levels was determined for a handgripping task through low-resolution electromagnetic tomography (LORETA) and a functional random effects approach (Wang et al., 2009). The time-dependent source strength function was reported to be nonlinear and to not vary significantly according to force levels. In another study, participants performed repetitive grasping movements at three different velocities and with four motor loads (Nakayashiki et al., 2014). The study reported significant weakening of time-averaged mu and beta event-related desynchronization (ERD) during the hold condition. In comparison, there was no significant difference under different load conditions. The results suggest a correlation between a time differentiation of hand postures in motor planning and ERD strength level. These investigations from a temporal perspective and their findings may substantially affect the design of improved tasks in the workplace.

When participants were required to apply 70% of their maximum voluntary contraction (MVC) in an isometric grasping task, the EEG and EMG data indicated that the electro-cortical and movement potentials increased with muscle fatigue (Johnston et al., 2001). However, coherence values decreased with the application of 50% MVC (Abdul-Latif et al., 2004). Another study using only 20% MVC in the isometric gripping task demonstrated that cortical activity increased in the preparation phase, peaked during onset, and then decreased thereafter (Yang et al., 2011). A synchronization was also observed among major sensorimotor areas. Halder et al. (2005) performed a microstate analysis after their experiment in which the participants engaged in the controlled repetition of a simple gripping task, i.e., squeezing a ball. With the application of an ERP technique, the study demonstrated a rapid change in cortical activity and distinct patterns at different stages of information processing (e.g., movement preparation, execution, and feedback integration) despite consistency in the effort and performance. When there was a cue, the preparation and execution of gripping tasks correlated with beta power (Zaepffel et al., 2013). An increase in beta power has even been observed at the start of the cue. In contrast, beta power has been found to decrease when participants prepare and perform a grasping task. While the object is held, there is a transient power increase as well. The results of beta power modulation have been found to be essentially consistent for two different types of forces and grips.

Fatigue is one of the effects of any continuing physical or mental activity, and this dimension is one of the major concerns of neuroergonomics. Some of the works here considered the fatigue aspects in their experiments. For gripping tasks, no significant variation in EEG power, amplitude, or MRCP negative potential (NP) was reported during the movement preparation phase despite a high fatigue level (Liu et al., 2005b). However, a significant decrease in power with fatigue was observed in the later sustained contraction phase. A follow-on study by Liu et al. (2007) further demonstrated that fatigue due to prolonged MVC in hand-gripping does not alter overall brain activation levels for controlling muscular movement. An estimation of single dipole locations has revealed a fatigue-induced shift of the brain activation center during a motor task. The shifts during the fatigued motor task were observed toward the right hemisphere and anterior and inferior cortical regions. Findings from the studies by Liu et al. (2007) have suggested the existence of alternating motor centers that compensate during fatigue and retain an optimally formatted output. For similar handgrip tasks, fatigue can be detected on the basis of a decrease in peak alpha frequency (PAF) (Ng and Raveendran, 2007). In a later study by Ng and Raveendran (2011), a dynamometer was used to create a task employing both hands. Increased theta and beta frequencies in different cortical regions were observed here. An interesting finding during muscular fatigue was the significant decrease in EEG-EMG coherence in the beta band despite an increase in power (Yang et al., 2009). However, Liu et al. (2005b) did find a decrease in power with muscle fatigue. This latter result may be due to the differences in the methodology and the equipment used in these studies. Additionally, the order of nonlinear operations such as artifact correction can affect the overall results of most EEG experiments (Luck, 2014).

A more recent study demonstrated significant EEG-EMG coherence during active exercise, i.e., grasping movements in different modes (Kim et al., 2017). These findings apply especially to the rehabilitation of stroke patients in verifying the presence of movement intention. Another recent work by Schwarz et al. (2018) showed that human MRCPs can significantly differentiate between reach-and-grasp tasks with regular objects versus a no-movement comparator. Furthermore, that study also demonstrated that EEG correlates in primary motor cortex can detect peak performance time, approximately 800–1,200 ms after the onset of movement. Investigators anticipate that these results can help neuroprosthetic technologies to restore function. A comparative study of EEG task-related power (TRPow) between older and younger adults by Hübner et al. (2018) demonstrated interesting effects on alpha and beta frontal power. Older participants showed less alpha frontal power at rest than their younger peers. However, during rest periods, the beta power of older individuals was greater than younger participants in multiple regions, i.e., frontal, central, and parietal. These results suggest that older adults need more power in the parietal and occipital areas while performing grip-based force modulation tasks. The study also demonstrated the necessity of controlling EEG power at rest to analyze TRPow when there are major age differences across participants.

In addition to EMG, functional magnetic resonance imaging (fMRI) is also used, as by Sclocco et al. (2014). Sclocco et al. proposed an EEG-informed fMRI analysis by modeling the changes in the spectral profile for different types of rhythmic brain activity. Here, participants performed a motor-oriented gripping task in active and resting phases. The proposed model was compared with other frequency-based regression models for EEG-fMRI data. Better results were found regarding the correlations between blood oxygen level-dependent signal and alpha and beta activity from EEG. This is an interesting observation and encouraging for similar studies on other forms of physical activity.

Nonlinear methods of EEG data analysis have also been reported. Here, Liu et al. (2005a) demonstrated that, for the gripping task, the fractal dimensions of the planning and preparation period do not correlate with handgrip force. However, the fractal dimensions of the moving and holding periods showed a positive correlation with handgrip force. In addition, a nonlinear parameter specified as the Lyapunov exponent (L1) was found to have larger values when the handgrip force is higher and to decrease significantly when participants approached fatigue (Yao et al., 2009). Thus, L1 has a statistically significant correlation with muscle force and fatigue, which can be utilized as a predictor of motor control-related cortical potentials.

#### Wrist Exertions

The first ever recording of MRCP was conducted on voluntary wrist movement by Bates (1951) with a photographic superimposition method. The study found that alpha activity was correlated with voluntary movement. More recently, beta and gamma band activity from EEG signals were investigated by Amo et al. (2016). They conducted an experiment where participants either rested or performed wrist bending tasks. The gamma index was calculated by using the power spectral density (PSD) in motor and basal areas. Of note, the study considered only central Cz channel data in order to avoid the significant artifacts associated with muscular exercise. The cited authors emphasized using their proposed gamma index as an indirect parameter to detect the activation of motor circuits. In isometric wrist exertion, Divekar and John (2013) demonstrated lower peak beta CMC, peak frequency, EMG beta power, perceived difficulty, and higher MVC and precision at the time of wrist flexion. An opposite pattern of results was found for wrist extension. However, for this latter comparison, there was no significant difference between power in the alpha and beta bands.

Furthermore, in relation to isotonic wrist flexion, nonlinear and linear cortico-muscular couplings were investigated (Yang et al., 2016). A generalized metric termed the “n:m coherence” was applied here to calculate nonlinear cross-frequency and linear iso-frequency couplings. Significant differences were reported in linear couplings between differing cortical regions. A stronger coherence was demonstrated for the primary sensorimotor areas and motor association cortices, i.e., the supplementary motor area (SMA) and prefrontal area, as compared with the sensory association area, i.e., the posterior parietal cortex. These results suggest that corticospinal tracts affect linear cortico-muscular couplings. In contrast, a nonlinear coupling has been found to originate from the sensory feedback pathways. Recently, using a wrist joint manipulation task, a nonlinear approach successfully correlated to cortical signals using a nonparametric formulation. Vlaar et al. (2018) used truncated Volterra series to develop models which revealed high-pass behaviorcorresponding to the high- frequency velocity signals from the muscle spindle to the cortex. The model also found a 46% relation between joint manipulation and cortical potentials without any prior assumptions. Another profound finding was that these results were consistent across each of the participants.

#### Hand and Arm Movements

One early investigation by Krause et al. (1983) focused on isometric exertion involving arm muscles, whereby peak alpha frequency was significantly higher when the participants performed the task with closed eyes. This was an interesting finding at that time, which could not be explained by the authors. Later, Oda et al. (1996) investigated with subjects performing isometric arm exertion with 10% and 50% of MVC. For the 50% force, frontal and central (Fz and Cz) areas were more active in terms of MRCP. Thus, force-dependent differences in neural activities reflected motor preparation and initiation. Others found more activity in all electrode locations (C3, C4, and Cz) when there was isometric elbow flexion with 20% MVC after the movement started (Shibata et al., 1997). The results were similar in three different cases, where one involved arterial occlusion during the exertion. MRCP values from the sensorimotor cortex and supplementary motor area were also found to be strongly correlated with isometric elbow flexion force, rate of increase in force and EMG signatures (Siemionow et al., 2000).

Most recently, Li et al. (2018) used the brain functional network (BFN) and hierarchical linear model (HLM) to decode neurophysiological signals for voluntary hand movement. The hand movement was performed following both straight and spiral trajectories. Delta, theta and gamma1 wavebands from eight electrodes in the frontal, central and temporal regions were found to be most sensitive to detecting associated effects. Previously, Wang et al. (2017) had investigated EEG signatures for three different force loads on the forearm, in a random order, until exhaustion. The results demonstrated a smaller alpha power for 1 and 3 kg weights compared with a no load condition. No significant differences were found between the latter two discrete load levels. Furthermore, a significantly greater alpha power was found when the participants were tired than when in no-fatigue control conditions.

#### Lower Limb Movements

Some reports have investigated lower limb muscular activities in addition to upper limb muscle activities. Beta and gamma coherence have been reported during static and dynamic force output, respectively (Gwin and Ferris, 2012). The participants in this study engaged in isometric and isotonic, knee, and ankle exercises. A major finding was the correlation between active muscular movements and corticospinal activities. Isometric tasks were found to be related to beta activity, whereas isotonic tasks were linked to gamma activity in the cortex. The results also demonstrated that a shift from beta to gamma is possible, depending on the muscle dynamics and proprioception. Another study measured the relation between beta coherence and force steadiness when the participants performed isometric ankle dorsiflexion (Ushiyama et al., 2017). The CMC was found to have positive correlations with beta band EMG signals and force fluctuation capacity.

### Physical Activity With Cognition

**Figure 6:**
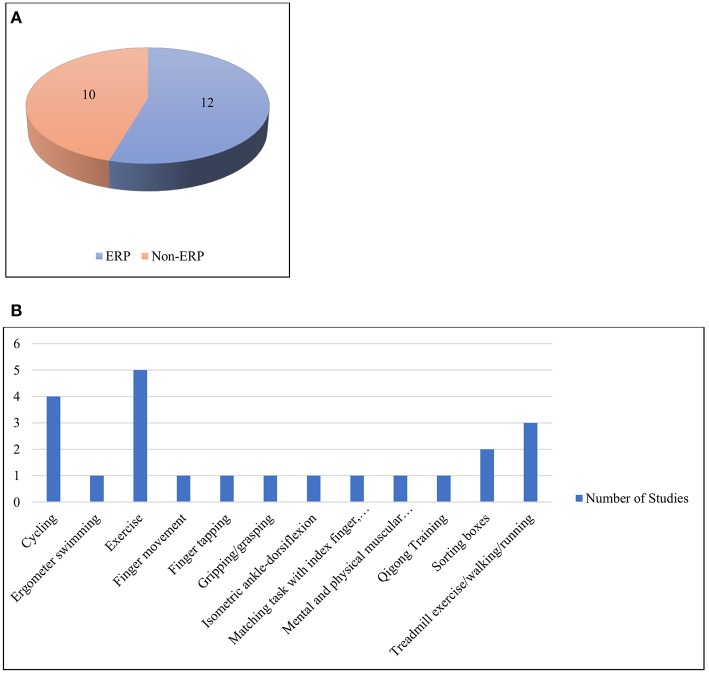
**(A)** ERP and non-ERP studies **(B)** Different types of physical activities under the category of physical activity with cognition.

One of the great shortfalls of modern behavioral science has been the nominal “separation” of mind and body (Marras and Hancock, 2013). Thus, biomechanics and physical ergonomics often treat the body solely as a physical entity, neglecting cognition. Similarly, many neuroscientists examine and evaluate the brain as though it were independent of the body that houses it. The growing consensus is that such separations are not simply unfortunate, but rather, actively misleading (Marras and Hancock, 2013). Consistent with our present neuroergonomic emphasis, we here explicitly consider published studies (n = 22) with participants engaged in tasks having both physical and cognitive demands. The works are summarized in Supplementary Table 2, Table 3 and visualized in Figure 6. In some of these studies, the participants performed both physical and cognitive tasks simultaneously, whereas in others, the tasks were performed either serially, cumulatively or interchangeably. Due to the heterogeneity of the works, they were divided into two broad categories based on the usage of ERP signals, i.e., those that were ERP-based and those that were not.

#### ERP-Based

ERPs are time-locked brain responses (Sur and Sinha, 2009). In general, having familiarity with a task before performing it is a major method to increase response efficiency. Thus, brain activity reflects the effect of mental practice (MP) on physical activity before task execution. An ERP study on the MP of physical activity has suggested that it has a positive effect on neuronal activities and performance (Allami et al., 2014). This study compared amplitudes and latencies of ERPs between two groups of participants, one only performing the grasping task and another learning the task by MP. The results demonstrated a rapid increase in ERP activity in the premotor cortex to reach the same amplitude for the group that performed both MP and physical execution.

Contingent negative variation (CNV) is an ERP component that shows a large negative peak deflection when a person is expecting a target stimulus (Luck, 2014). Lang's bioinformational theory (Lang, 1979) has been applied to three groups of participants by combining those who had to perform tasks both physically and mentally (Smith and Collins, 2004). The first study included MVC of the hand muscles, and the second included MP of a virtual task of knocking down a barrier with an associated cognitive challenge. In the first study, late CNV waves were observed for all participants. However, in the second study, late CNV was not found for the stimulus-trained group. The study results support Lang's theory of cognitively oriented motor tasks.

The P300 is one of the most studied ERP components, so named because it peaks at approximately 300 ms. The EEG-EMG co-registration method has been applied by De Tommaso et al. (2015) when participants performed sitting, standing, walking, and a P300 oddball paradigm while standing and walking. The amplitude of the P300 increased in the presence of physical activity. Alpha rhythm was found to be reduced in spectral width during a cognitive task performed while standing. In comparison, the alpha rhythm disappeared when there was movement without any cognitive activity. Additionally, mu-ERDs were found in 60% of the steps taken. However, another study demonstrated a different result, i.e., that the P3 component did not increase when there was physical activity (Killane et al., 2013). This experiment included four segments: control, static, bicycle, and treadmill, along with an auditory oddball task. In all four cases, the P3 peak amplitude and latency did not show significant variation.

P1 is another major ERP component with sensitivity to the variations in stimulus parameters. It is largest in the occipital region (Luck, 2014). An interesting finding has been observed in participants performing high-intensity exercise. Target detection in this case proved to be faster than that during rest and low-intensity exercise (Bullock et al., 2015). The mean amplitude of the parieto-occipital P1 component has been reported to be larger during low-intensity exercise than a resting phase. However, during low-intensity exercise, the P1 component increased significantly compared with rest and high-intensity exercise. In addition, the peak latency of the parietal P3a component decreased as a function of both low- and high-intensity exercise.

In our survey, we found studies that investigated the relationships between the fitness of a person and their EEG results, ERP components, and cognitive and physical performance. An early ERP study analyzed the electrophysiological effects of fitness and maximum aerobic exercise during cycling (Magnie et al., 2000). Participants were divided into groups according to their fitness level and given cognitive tasks before and after the exercise. No significant difference was found in the ERP components of P300 and N400 across different fitness levels. Furthermore, the amplitudes for both components and latency for P300 increased and decreased, respectively. The P3 or P300 component of ERP is thought to be involved in updating the context and working memory (Donchin and Coles, 1988). In addition, the results were applicable when the participants returned to a normal state and were not fatigued due to exercise. A later experiment demonstrated that cardiorespiratory fitness had a positive effect on cognitive performance (Themanson and Hillman, 2006). Error-related negativity (ERN) amplitudes during a Flanker task were lower for the fitter adults. However, no such effect was observed on cognitive processes after 40 min of intense exercise on a treadmill.

Chang et al. (2015) investigated participants performing acute exercise and the Stroop test according to their fitness condition. They recorded ERD and found that exercise helped improve cognitive performance in the form of greater alpha ERDs. In contrast, there was no significant change in the lower and upper alpha ERDs corresponding to cognitive performance and fitness. However, in a later experiment, Chu et al. (2016) demonstrated that cognitive function is affected by the level of cardiorespiratory fitness, thus confirming the findings of the study by Themanson and Hillman (2006). In another experiment, trained athletes performed both prolonged cognitive and control activities and then a complete endurance exercise of cycling (Van Cutsem et al., 2017). Interestingly, the mild fatigue from the cognitive task did not affect the physiological or perceptual metrics. The EEG results demonstrated increases in theta and alpha waves and P3b latency, as well as a decrease in P3b amplitudes, thus confirming the high cognitive demand of the task.

Despite most of these investigations being focused on exercise and simple physical activity, others have evaluated workplace jobs, e.g., box sorting along with a cognitive task (Wascher et al., 2014). The major findings here include a reduction in the posterior P3 component during physical task performance. Furthermore, the fronto-central N2 component and theta activity increased when the participants solved a Sudoku puzzle, i.e., a cognitive task. Later, Wascher et al. (2016) altered the design of the study by replacing the Sudoku with two types of cognitive tasks: monotonous and self-paced. Increased error rates and alpha activity were reported in monotonous cognitive tasks in younger participants showing mental fatigue. In comparison, inefficient information processing was observed for older subjects in the form of varying EEG signals, time-locked to blinking.

#### Non-ERP-based

We also evaluated works that did not explicitly consider ERPs in their data recordings or in later processing stages. Most of these studies measured PSD, wave amplitudes, feature selective profiles, and source reconstruction in the respective data processing stages.

As with the prior section, a significant number of investigators here focused on MI and MP along with the actual execution of the physical task. In a study by (Blinowska et al., 2010), finger movement tasks, designed with different levels of complexity, tended to provide consistent cortical signal information. In particular, for a simple task such as pressing a button, bursts of EEG have been observed in the associated areas of the motor cortex as well as in the frontal cortex. Alternatively, if participants are given a more difficult task, such as imagining such a movement, the sensorimotor areas become more active. The most difficult task among the investigated studies was the continuous attention test (CAT), in which the index finger had to be moved according to specific instructions. Here, the prefrontal cortex (PFC) was most engaged. A recent work showed that motor execution and imagery are dependent on inputs to the premotor cortex (PMC) and SMA, respectively (Kim et al., 2018). In addition, coupling between PMC and the dorsolateral prefrontal cortex (DLPFC), i.e., DLPFC-PMC, was higher when there was a correct imagery response. For an incorrect MI response, PMC-SMA coupling was more pronounced. EEG activities have also been investigated by Henz and Schoellhorn (2017) in participants engaged in physical and MP of the stress-relieving physical exercise called Qigong. There was an increase in the alpha and theta activities in the posterior and fronto-central areas. Interestingly, similar effects were observed in the MPs.

EEG signatures have also been investigated without concomitant consideration of MP. Mierau et al. (2009) divided participants into three groups on the basis of the task to be performed. These included a running group (RG), a tracking group (TG), and running followed by tracking group (RTG). Smaller tracking errors indicated better task adaptation in RTG than TG. Furthermore, EEG recording after tracking demonstrated increased alpha and beta power in TG as participants were cognitively aroused. There were no incremental alpha or beta activities in RTG, thus indicating a relaxed and somewhat inactive state of the brain. In addition, since there was no change in the spectral power in RG, the observed difference between TG and RTG was not due to physical recovery. Instead, it was due to the cortical efficiency and exercise-induced capability of selective central processing according to the actual task requirement. Additionally, mental fatigue from a previously performed Rapid Visual Processing (RVP) test was demonstrated by increased theta power after 20 km cycling (Pires et al., 2018). Different PFC activation for RVP and cycling, deteriorated performance and increased RPE were also the indicators of mental fatigue. Among the works involving EEG and EMG, Shinohara (2014) engaged participants in motor-oriented and cognitive tasks in different blocks in between an active leg resistance task. After the exercise, there was a decrease in EEG oscillations and beta corticomuscular coherence. Other major findings here included (1) a significantly negative correlation betweenbeta coherence and the EMG variation coefficient (2) greater perceived exertion and physiological stress after the assigned leg exercise.

A more recent study concerning both cognition and exercise together, applied an inverted encoding model (IEM) to extract feature-selective response profiles from EEG data (Bullock et al., 2017). The participants completed an orientation discrimination task while riding an exercise bike. An interesting finding of this study was that low-intensity cycling corresponded to the highest gain in response profiles. The overall findings support the proposal that the human visual cortex is more sensitive during active movement. Neurofeedback helps trained individuals gain some degree of control over electrophysiological processes (Demos, 2005). For example, Mikicin and Kowalczyk (2015) demonstrated a decrease in beta amplitudes along with a reduction in reaction time in both an attention-reaction test and the Kraepelin test after 20 neurofeedback sessions. Participants performed the Kraepelin curve test, an attention-reaction computer test, and neurofeedback-EEG training sessions while performing submaximal exercise. The reduction in the amplitudes of beta activity was reported to be effective in improving the attention task performance. This study supported the contention that neuro-feedback-EEG training in motion can be an effective method for improving work performance. When traction exercise was performed with visual feedback, a cyclic pattern in theta frequency was observed by De Hillerin et al. (2015). The participants here engaged in a series of mental and physical exercises, along with a relaxation phase. Different phases demonstrated different types of cortical activity, along with the evolution of theta, alpha, and beta frequencies.

Investigations have also been conducted regarding neural responses when there is a change in cues or the presence of distractor stimuli. Bigliassi et al. (2018) found that auditory stimuli, i.e., music, may work as a distractor when there is a cognitive-motor task of high demand to be performed (and see Szalma and Hancock, 2011). In this case, the task was isometric dorsiflexion where participants had to monitor the length and intensity of their contractions. The results showed active right and central parietal regions of the brain after the auditory stimulus was initiated (around 0.368 s). From source reconstruction analysis, this may reference the brain's inhibitory signals toward the processing of task-irrelevant stimuli, i.e., the auditory distractor.

### Miscellaneous Physical Activities

**Figure 7:**
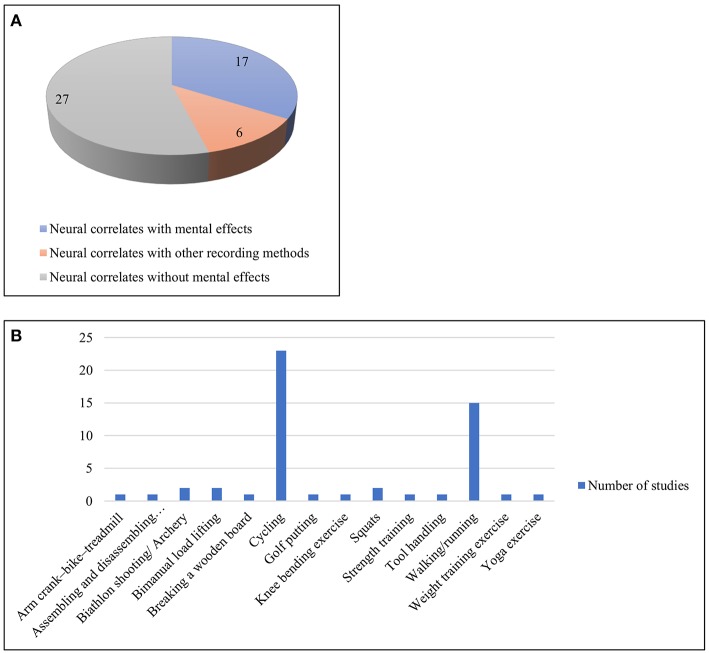
**(A)** Three sub-categories of works with miscellaneous activities **(B)** Different types of physical activities under the miscellaneous category.

This category of papers (*n* = 50) included a wide variety of tasks ranging from walking, running, cycling, yoga, and swimming to shooting and other exercise and sporting activities. However, walking and cycling were two of the most studied physical activities here. The works have thus been divided into three broad sections, based on their consideration of mental effects, perception and use of other recording methods along with EEG. These works are summarized in Supplementary Table 3, Table 4 and visualized in Figure 7.

#### Neural Correlates Without Mental Effects

In one of the earliest of these studies, Daniels et al. (1984) examined runners and bikers. They found a significant increase in alpha band activity in both hemispheres. In the case of runners, the increase was mostly during the last 10 min of exercise. However, a decrease in alpha band was observed while approaching the maximum intensity experienced. A later study demonstrated that when cycling, participants' beta amplitudes in the occipital midline regions were inversely correlated with their physical condition, as measured by Vervaeck's Index (VI) (Kakizaki, 1988). Other early studies also exhibited similar results, e.g., Beyer et al. (1989) who showed increased alpha activity after exercise in parietal and occipital areas. A later work by Collins et al. (1990) involved karate performers whose alpha activity was higher immediately before breaking a wooden board. In addition, the brain was demonstrated as being more active when compared between exercise and non-exercise conditions (Kubitz and Mott, 1996). Alpha activity decreased and beta increased during exercise, i.e., cycling. However, in a later study, Kubitz and Pothakos (1997) found different results, i.e., lower levels of brain activation with increased alpha and theta and decreased beta activities.

Early studies also worked with other forms of exercise such as repetitive knee bending exercise, yoga, and others. Gotze et al. (1966) recorded EEG signals with telemetry, and the results showed less brain activation and decreased frequencies during hyperventilation. The participants were engaged in 20-50 knee bending movements. Dostalek et al. (1980) demonstrated unique, sharp patterned and reproducible waves during hatha-yogic exercise.

Bailey et al. (2008) distinguished a significant increase in all major frequency bands of EEG after a gradually intensifying cycling exercise. This phenomenon is, to a degree, stable across all electrodes, also showing no significant difference between hemispheres. A recent study has also shown that, in addition to cycling, moderately intense running significantly increases theta, alpha, and beta amplitudes (Choktanomsup et al., 2017). However, these results also showed a decrease in alpha activity and overall spectral power in the brain when the participants were involved in a task requiring high aerobic demand (Ludyga et al., 2016). In particular, this phenomenon was more pronounced when pedaling frequencies were high. This finding supports those from other studies, e.g., Liu et al. (2007) regarding the ability of the brain to adapt and compensate at the time of high-intensity cycling, i.e., high muscular activation, which ultimately improves endurance performance. Ftaiti et al. (2010) investigated the changes in the alpha to beta ratio during prolonged and high-intensity cycling. They reported that the ratio changed more when the participants were working in a hot environment than when the exercise intensity and temperature were lower (see López-Sánchez and Hancock, 2019).

Spring et al. (2017) have conducted EEG microstate analysis of endurance-trained participants who performed two types of biking. Microstates are determined on the basis of four conventional map topographies (Koenig et al., 2002). The stability and duration of a particular microstate C increased during the rest state before and after the exercise. Furthermore, the correlation between the increase in C duration and decrease in maximal voluntary force indicated that the resting state motor cortex activity was associated with the motor output (and see Hancock, 1981, 1982).

Human gait pattern is another complex motor coordination task in which the brain plays a major role. For example, at the moment when there is a change in direction between flexion and extension, the cortical activation is highest (Wieser et al., 2010). Presacco et al. (2011) further observed highly active fronto-posterior cortical regions in both normal and precision walking. Changes in PSD, corresponding to the gamma band, were observed by Gwin et al. (2011) in between steps, while they were studying steady-state gait. These changes were particularly expressed in the anterior, posterior parietal and sensorimotor cortex areas.

A later study collected data from EEG electrodes and an accelerometer simultaneously during treadmill walking (Castermans et al., 2014). Maximum motion artifacts up to 15 Hz were reported, including the stepping frequency of the participants. This finding raises questions regarding the previous investigations, e.g., Presacco et al. (2011), who reported a fundamental stepping frequency of approximately 1 Hz by using a band pass filter only. Furthermore, time-frequency analysis demonstrated similar and broad (up to 150 Hz) rhythmic activities for both EEG and accelerometers. In addition, Gwin et al. (2011) reported intra-stride high gamma activity via event-related spectral perturbation (ERSP) analysis as well as independent component analysis (ICA) only as a data cleaning tool. The investigators recommended a thorough and robust data cleaning method to study cortical activities during human locomotion (Castermans et al., 2014).

Other major findings, while studying walking, included a reduction in alpha and beta frequency over the electrodes reflecting leg motor regions. In addition, greater suppressed mu and beta ERDs have been found when there is a change in movement, such as upright walking (Seeber et al., 2014). A dynamic, low gamma amplitude modulation represents the gait cycle phase. Because the ERD and gait phase modulation center frequencies differed, their origins were deemed to be separate rhythmic activities. A significant difference in cortical activities has been reported between normal and stabilized walking (Bruijn et al., 2015). The difference was observed only in the left premotor area. That study has demonstrated higher beta power, indicating an active cortex during steady gait. Peterson and Ferris (2018) showed an active occipito-parietal and active sensorimotor area for two types of perturbations, i.e., visual and physical pull, respectively. The task included both standing and walking. During the initial phase of these modifications, theta synchronization and alpha-beta desynchronization were also observed.

In exercises such as isometric squats, in which only the resistance of body weight is used, no other equipment is needed. Several studies have investigated EEG signals with participants performing these forms of squat. Changes in MRCP were demonstrated in participants performing squats of different intensity (Comstock et al., 2011). The changes were recorded in the prefrontal, central, posterior parietal, and occipital regions. The generation of unique topographical maps has demonstrated MRCP for different types of resistance exercise, i.e., these forms of squat. Recently, for the first time, a study was conducted by recording EEG signals during bench press weight training (Engchuan et al., 2017). Here, a significant increase in beta and gamma amplitudes was observed at the time of intense exercises.

Some of the physical activities in the industrial setting are of the manual lifting type, or for assembly jobs, manual tool handling, etc. One study was designed to include two EEG analyses, time-frequency analysis and ERP analysis, when the participants engaged in a bimanual lifting task (Barlaam et al., 2011). Similar to the findings of another study with walking, there was a decrease in the mu power of motor cortices prior to the onset of the stimulus. Furthermore, in the ERP results, the left and right motor cortices demonstrated negative and positive waves, respectively. These results were interpreted as indicating that the negative wave was due to the upcoming lift, whereas the positive wave was due to the inhibitory command to the forearm itself. During a light assembly task, with low and high task loads, the mean alpha power values indicated the level of that load (Zadry et al., 2010). The mean alpha power in the frontal and parietal (Fz and Pz) regions was greater for a higher load. However, the values were higher in occipital channels (O1 and O2) for a lower load. These findings were taken as reflecting fatigue in the tasks with higher loads.

Motor-related neural activities at the time of manual tool handling and learning were investigated by Mizelle et al. (2011). Participants were exposed to direct physical practice and indirect video-based observation with both familiar and unfamiliar tools. For direct exposure, the unfamiliar tool pantomime involved greater activation in the parietofrontal areas of both hemispheres. In contrast, the left parietofrontal and right temporoparieto-occipital zones were more active for both familiar and unfamiliar tools, respectively. These crucial findings can help us to design better user-task interfaces, consisting of manual material handling. Another work on learning to putt with the intention of teaching showed better post-test performance than another group without teaching intention (Daou et al., 2018). EEG signals during the onset of the putt reflected reduced action monitoring, use of working memory, and enhanced motor programming. These were translated from a linear reduction of frontal midline theta and upper alpha power. Other results include increased frontal midline theta but decreased upper alpha power in both left and right areas during the periods of practice compared to pretest.

Salazar et al. (1990) worked on sport, i.e., archery, which demonstrated that the changes in EEG signatures in the left hemisphere reflect changes in shooting performance. Heart rate (HR) did not decrease and was not found as one of the indicators of attention in this scenario.

#### Neural Correlates With Mental Effects

As noted previously, the inclusion of mind and body together is one of the major themes of neuroergonomics (Parasuraman and Hancock, 2001; Marras and Hancock, 2013). A significant number of works have considered effects on moods, emotions, fatigue, preference and perceptions while engaged in a physical activity. Some of these investigations used music and other auditory stimuli to learn about their moderating influences. For instance, an early study by Wales and Thayer (1985) found a more active left hemisphere in the brain when the participants were listening to fast, positive music and cycling simultaneously. However, this was not the case for fast, negative music. Changes in RPE were not significant either. The perceived exertion tended to decline when participants performed low to medium intensity exercise in a cycle ergometer while listening to music (Bigliassi et al., 2017). Listening to an audiobook increased alpha activity in the central zones, owing to semantic and perceptual processing. However, the results related to music indicate decreased focal awareness, halted alpha resynchronization and more efficient motor control.

Stress due to adverse environments (Hancock and Warm, 2003) was the focus of several works. An early study reported that perceived exertion correlated more with EEG than EMG after cycling in a hot environment (Nybo and Nielsen, 2001). Hyperthermia due to cycling exercise may slow down EEG signals, increase RPE, and decrease cerebral perfusion (Goodman et al., 1984; Rasmussen et al., 2004). Moreover, EEG changes could not be causally linked with the decrease in cerebral perfusion, as the manipulation of the latter did not affect EEG or RPE. It has been suggested that fatigue due to increased brain and core temperatures may be a reason for EEG changes. Recently, Périard et al. (2018) demonstrated that alpha and beta activities in frontal and central areas decreased when the participants cycled in a hot environment. In the case of hypoxia, alpha activity in these regions was lower than a comparative control. However, beta was not substantively different as compared to the control and higher than hot conditions. Here, these changes in frontal and central areas helped meet the challenge of attention management and arousal in a stressed environment.

The change in neural connections due to fatigue after cycling was first investigated by Hilty et al. (2011). A significant increase in lagged phase synchronization between the mid/anterior insular and motor cortex was observed at the end of cycling. Tuncel et al. (2010) investigated physical fatigue during weight lifting by applying time-frequency coherence analysis. Their study consisted of three stages of fatigue and demonstrated a significant decrease in coherence at the last stage compared with the other two time-frequency domains. Flanagan et al. (2012) divided participants into three groups according to the rate, magnitude, or volume of force in a squat type resistance exercise. Fatigue and cortical activity amplitudes were observed to be proportional. Furthermore, the volume phase of the task demonstrated the largest amount of increases in cortical, motor, and sensory activities despite having non-incremental and reduced loads.

Moraes et al. (2011) assessed the changes in EEG power and participants' mood after cycling in two distinct age groups. In younger participants, a significant improvement in mood (vigor and anger) and changes in alpha and beta activities were observed. The total mood disturbance improved for both age groups, and a positive correlation was reported between frontal alpha asymmetry and mood disturbance in older participants. The correlation was negative in the younger participants. For young female participants, greater frontal left activation and a robust change in frontal alpha asymmetry were observed after acute exercise than after rest (Woo et al., 2010). These findings also indicated similar affective responses at the time of recovery after participants performed the steady-state aerobic exercise of different intensities. Another study was conducted on adolescent participants and demonstrated different affective responses after the participants exercised with different intensities (Schneider M. et al., 2009). When the participants primarily performed moderate cycling without being informed of the intensity of the task beforehand, frontal cortical asymmetry correlated with affective response. Also, affective response was more positive for left-handed participants than right-handed participants. In contrast, when they expected a high-intensity task but then were asked to do otherwise, there was no difference in affective responses of the left- and right-dominant participants. Moreover, no significant correlation between frontal asymmetry and affective response was observed in this case. Due to the absence of difference when there is an expectation of an extreme physical task, it can be interpreted that the influencing mechanism of cortical asymmetry on the affective response is more applicable for cognition than physical activity.

Cortical activity also indicates exercise preference (Schneider et al., 2009b). In an experiment by Schneider et al. (2009b), the participants were involved in three types of exercise, i.e., treadmill, bike, and arm crank ergometry. Here, they expressed a preference for running. An increase in frontal alpha activity was observed after treadmill exercise only. Because the frontal alpha activity has an inverse connection to cerebral activity, it was assumed to be linked with preference. The study demonstrated an increased alpha activity in the parietal region after biking and increased beta activity in the Brodmann area after all three exercises.

In addition, task duration was reported to be a factor as separate patterns of cortical activities have been observed after approximately 15 and 30 min of exercise, respectively. Schneider et al. (2009a) conducted an experiment in which the participants ran on a treadmill at three different intensities: low, preferred, and high. The results suggested that both EEG activity and mood are affected by preferred and high-velocity exercise. The study also demonstrated correlations among perceived physical and motivational states, psychological strain, and alpha and beta brain activities. Other studies focused on the affective responses of treadmill exercise at three different intensities of ventilator threshold (VT) (Hall et al., 2010). A significant resting mid-frontal EEG asymmetry was demonstrated to predict affective state (i.e., energetic and tense arousal) at below-VT intensity level. Similar results have been observed in the above-VT condition. In particular, the study had an opposite finding from its hypothesis, i.e., when there is an increase in relative left frontal activity in the brain, energetic arousal is lower.

Along with EEG, investigations have measured feeling scales, felt arousal scales, RPE, and heart rate before, during, and after preferred or prescribed exercise (Lattari et al., 2016). There were greater values of heart rate and RPE for prescribed exercise (PE) and self-selected (SS) conditions than for controls. From the perspective of feeling scale, these values were higher for SS than PE and controls. For the other scale, i.e., the felt arousal scale, both PE and SS showed higher values than the controls. However, in contrast to the other studies, this study found no changes in frontal alpha asymmetry for both exercise types, even though SS provided better affective responses. Moreover, there was no interaction between condition and moment for frontal alpha asymmetry (FAA). Other studies investigated how FAA can be linked to emotional states. Recently, Hicks et al. (2018) conducted a study to investigate whether FAA is causally related to cardiovascular demands or the movement required for a certain task. The study included two types of physical activities along with a control group. The results support those from previous studies by showing an approximately 20 min delay in increasing FAA. However, the significant increase in FAA was observed in aerobic exercise only, which required a cardiovascular load. In contrast, there was no significant change in FAA for the task of bilateral movement, thus not supporting the hypothesis. Therefore, the tasks imposing a substantial cardiovascular load could cause an increase in FAA and eventually the motivation level. However, to address the question of Hicks et al. (2018) as to why there was no change in FAA after SS, further investigations are required.

Beyond typical physical exercise, there are investigations into sports such as shooting. For biathlete shooters, HR and RPE increased with cardiovascular load (Gallicchio et al., 2016). In EEG power spectral analysis, a decrease in pre-shooting frontal-midline theta power and increase in temporal and occipital alpha power were observed. A higher accuracy was reported in the presence of a higher frontal-midline theta, lower left-central alpha, and higher left-temporal alpha power. Greater inhibition of movement-irrelevant regions (temporal, occipital) and activation of movement-related regions (central) indicated that greater neural efficiency is beneficial to shooting performance despite physical loads. For all three works, a common observation was an increase in alpha band activity.

#### Neural Correlates With Other Recording Methods

Along with EEG, other psychophysiological recording methods such as EMG and electrocardiography (ECG) have been used to investigate physical activities. A study by Storzer et al. (2016) comparing EEG and EMG indicated that cycling and walking induce different degrees of cortical activation. The EMG data showed a cyclic power modulation within the range of 24–40 Hz for both tasks. In the EEG data, biking was associated with stronger and sustained cortical activation, a beta power decrease at the time of movement execution, and less cortical motor control at the time of the movement cycle. However, walking was associated with a stronger and sustained power decrease in the alpha band. Furthermore, in multi-terrain gait analysis and EEG-EMG coherence analysis, EEG activity precedes EMG activity in the low gamma band in the phases of swing and stance for both ground level walking and ramp ascent, respectively (Winslow et al., 2016).

There are other forms of physical exercise focusing on muscular strength (Hoff et al., 2002). The load levels are higher for strength training (ST) than endurance. To build up endurance, the load is lighter with more repetitions. Cortical activity when performing these forms of exercise was investigated by Dal Maso et al. (2012). This study was also the first to demonstrate cortical adaptations associated with an ST task. For the ST participants, suppression in the beta band of 21–31 Hz increased with the force level. In addition, less activation of the antagonist's muscles was observed in ST participants than endurance discipline (ED) participants on the basis of EMG signals. In a more recent study, Dal Maso et al. (2018) demonstrated a reduction in beta ERD over left sensorimotor areas and an increase in alpha and beta functional connectivity over the left and right sensorimotor areas, as well as an increase in the CMC in a few electrodes. However, there was no correlation between CMC and motor skill retention. This work included participants performing a single bout of intense physical exercise, in this case, cycling. Simultaneous recordings of EEG and EMG signals were also conducted when the participants were engaged in isometric and dynamic exercises (Zhang et al., 2011). In regular exercisers, MVC, RMS, and EMG parameters are higher than in people who scarcely exercise. These authors suggested that motor neurons of regular exercisers mobilize more quickly than those of their sedentary peers.

In addition to EMG, EEG along with ECG have been applied by investigators. Lin et al. (2017) recorded both ECG and EEG patterns and investigated how an increase in the average-to-maximal heart rate ratio (AMHRR) affected brain activity during continuous cycling. Two parameters, EEG spectral power and fuzzy entropy (FuzzyEn), demonstrated similar increasing patterns with AMHRR at all electrodes. However, a significant correlation between FuzzyEn and AMHRR was reported in only the alpha, beta, and theta bands. FuzzyEn also demonstrated high feature selection specificity and effective detection of changes in EEG patterns during exercise.

### Future Research

Each of the works discussed in this review contribute to explanations of brain activities in real time when a participant is engaged in or has just finished a task requiring physical movement. The collective work under the category involves light muscular activities including isometric exertion, moving the fingers, gripping or grasping, and moving the wrist and the lower limbs. All of these were conducted in controlled experimental environments. These movements are regularly performed in everyday life both at work and in realms beyond the workplace. For instance, using a computer keyboard, mouse or handheld devices are representative of the real world application of the finger, wrist and in some cases arm movement. Therefore, both replications of existing works and new exploratory studies are necessary where a real-life activity is performed in ecologically valid settings.

The second category of studies included physical activities with the concomitant use of cognition. Again, the majority of these works lack designs with ecological validity. Only one included a real-life industrial task, i.e., sorting boxes. Similar activities should be studied where people use both physical and cognitive skills, e.g., manual assembling and disassembling, manual inspection of products, etc.

Finally, works grouped under the miscellaneous category here included cycling, walking and physical exercise of other forms as well as sporting activities. Although there are works on lifting, other activities related to manual material handling, e.g., pushing or pulling, rolling, or moving items, are less explored. One of the concerns of these works was consideration of the perceptions and mental effects along with neural correlates due to a physically demanding activity. Earlier, Gielo-Perczak and Leamon (2002) demonstrated that participants engage in risky overexertions beyond their capacity when maximum effort is encouraged or enforced. Therefore, the continuation of similar work considering perceptions, emotions and other mental effects with unexplored activities at work promise to be essential.

In the scientific world, there is a considerable data gap with respect to females (Perez, 2019). Although there are works including both male and female participants, there are not many such studies that concern physical activities performed by females alone. Research has shown a significant difference in strength between males and females (Maughan et al., 1986). Hence, replication and comparison of EEG studies between males and females is another gap to be closed by further research.

## Conclusion

In addition to considerations of cognition alone, neuroergonomics investigates substantial information regarding brain signatures in relation to human physical performance (Hancock, 2019). The present literature review has categorized and extracted key findings from EEG experiments in 110 selected works. Furthermore, information concerning different aspects of EEG experiments, e.g., the type of tasks, subjects, recording electrodes, approaches to artifact correction, and data processing methods, have also been included. The review can be considered a focused follow-up to the previous review by Mehta and Parasuraman (2013). However, a comparative study of all results from each study was beyond the scope of one literature review. Reporting the quantitative statistical results of each study was also not possible due to their evidently inhomogeneous nature.

The results of this review serve to answer the present research questions and demonstrate the substantial role of brain activities in controlling performance, fatigue, preference, emotion, cognition, and perception in relation to physical movement. EEG signatures also have significant correlation with load levels, intensity, modes, stages of task preparation and execution. Investigators inspected major bands of rhythmic activities, e.g., theta, alpha, beta, and gamma. Alpha activity was primarily found when there was fatigue, cognition and less physical activity. FAA was found to be correlated with cardiovascular demand and affective responses. Beta and gamma were observed mostly during physical activities. Significant changes in gamma activity were detected at the time of walking. Differences in various ERP components such as P3 or P300, P1, N2, N400, CNV, and ERN were detected when there was physical activity with cognition. Brain areas, e.g., PMC and SMA, were found to be more active during motor execution and imagination, respectively. Correct and incorrect imagery are also distinguishable through neural activities. In addition, parameters associated with other recording methods, e.g., BOLD, EEG-EMG coherence, and AMHRR were correlated with EEG signatures in some investigations.

In the current review, research gaps were also identified, and recommendations have been suggested for future study. We found a lack of research in naturalistic settings and few comparative and replicative efforts based on sex. Additionally, most of the studies relied on visual/manual inspection for artifact correction and made limited use of other tools. Moreover, the results of a few works were questioned or contradicted by others. Thus, more research is needed to clarify those cases of conflict. Accordingly, given the many unexplored and less studied areas regarding EEG, we believe that physical neuroergonomics has great research potential with increasing interdisciplinary and global collaboration among researchers.

## Author Contributions

MR conducted the literature search and prepared the initial draft of the paper. WK supervised all aspects of manuscript preparation, revision, editing, and final intellectual content. MR and WK were involved in study conception and contributed to intellectual content. PH and MF contributed to intellectual content and edited the paper.

### Conflict of Interest Statement

The authors declare that the research was conducted in the absence of any commercial or financial relationships that could be construed as a potential conflict of interest.
