# Corrigendum: Neuroergonomics Applications of Electroencephalography in Physical Activities: A Systematic Review

**DOI:** 10.3389/fnhum.2019.00277

**Published:** 2019-08-13

**Authors:** Mahjabeen Rahman, Waldemar Karwowski, Magdalena Fafrowicz, Peter A. Hancock

**Affiliations:** ^1^Computational Neuroergonomics Laboratory, Department of Industrial Engineering and Management Systems, University of Central Florida, Orlando, FL, United States; ^2^Department of Cognitive Neuroscience and Neuroergonomics, Institute of Applied Psychology, Jagiellonian University in Krakow, Krakow, Poland; ^3^Department of Psychology, University of Central Florida, Orlando, FL, United States

**Keywords:** EEG, neuroergonomics, ergonomics, physical exercise, physical activity, physical exertion, brain, human factors

In the original article, the reference for the in-text citation “(see Sanchez-lopez and Hancock, 2019)” was incorrectly written as “Sanchez-lopez, I., and Hancock, P. A. (2019). Diminished cognitive capacities in an ever hotter world: evidence from an applicable power-law description. *Hum. Factors*. doi: 10.1177/0018720818816436. [Epub ahead of print].” It should be “López-Sánchez, I., and Hancock, P. A. (2019). Diminished cognitive capacities in an ever hotter world: evidence from an applicable power-law description. *Hum. Factors*. doi: 10.1177/0018720818816436. [Epub ahead of print]”.

A correction has been made and the in-text citation should read “(see López-Sánchez and Hancock, 2019)”.

The authors apologize for this error and state that this does not change the scientific conclusions of the article in any way. The original article has been updated.
