# Supplementary material for: Neuroergonomics Applications of Electroencephalography in Physical Activities: A Systematic Review
Source: Front Hum Neurosci. 2019 Jun 4;13:182. doi: 10.3389/fnhum.2019.00182 (PMC6558147; doi:10.3389/fnhum.2019.00182)
Supplement: Supplementary file 1 [file Table_1.docx]

Supplementary Material

Neuroergonomics Applications of Electroencephalography in Physical Activities: A Systematic Review

Mahjabeen Rahman, Waldemar Karwowski*, Magdalena Fafrowicz and P. A. Hancock

*** Correspondence:** Waldemar Karwowski: wkar@ucf.edu

# Supplementary Tables

**Table 1.** Studies investigating muscular activity with less mobility

| Study No. | Authors | Subjects' gender | Physical activity | Data processing method/parameter | Citation No./Years after publication | Number of electrodes | Artifact correction |
| --- | --- | --- | --- | --- | --- | --- | --- |
| 1 | (Abdul-Latif et al., 2004) | M/F | Gripping/grasping | Coherence | 0.27 |  | Visual/manual correction |
| 2 | (Chakarov et al., 2009) | F | Finger movement | EEG-EMG coherence | 8.6 | 51-60 | Visual/manual correction |
| 3 | (Liu et al., 2005a) | M/F | Gripping/grasping | Non linear | 4 |  | Visual/manual correction |
| 4 | (Yao et al., 2009) | M/F | Gripping/grasping | Non linear | 1.6 | 61-70 | Noise source minimization |
| 5 | (Zaepffel et al., 2013) | M/F | Gripping/grasping | Time-frequency analysis | 7.67 | 61-70 | Visual/manual correction |
| 6 | (Gwin and Ferris, 2012) | M/F | Lower limb movement | Corticomuscular coherence | 6.29 | 251-300 | Channel rejection, AMICA, DIPFIT |
| 7 | (Kim et al., 2017) |  | Gripping/grasping | Coherence | 0 | 61-70 |  |
| 8 | (Wang et al., 2017) | M | Hand and arm movement | Feature extraction | 0 | 31-40 | ICA |
| 9 | (Yang et al., 2016) | M/F | Wrist exertions | Coherence | 2 | 101-150 | ICA, DIPFIT |
| 10 | (Negro and Farina, 2011) | M/F | Finger movement | EEG-EMG coherence | 8 | 31-40 | Visual/manual correction |
| 11 | (Johnston et al., 2001) | M | Gripping/grasping | Movement related cortical potential (MRCP) | 3.39 | 21-30 | Trend correction |
| 12 | (Slobounov et al., 2004) |  | Finger movement | Movement related cortical potential (MRCP) | 4.34 | 10-20 | Trend correction and Neuroscan software |
| 13 | (Wang et al., 2009) |  | Gripping/grasping | Low Resolution Electromagnetic Tomography (LORETA) | 1.7 | 61-70 | Visual/manual correction |
| 14 | (Feige et al., 2000) | M/F | Finger movement | EEG-EMG coherence | 7.11 | 61-70 | EOG signals correction |
| 15 | (Halder et al., 2005) | M/F | Gripping/grasping | ERP | 2.93 | 61-70 | ICA and algorithm |
| 16 | (Yang et al., 2011) | M/F | Gripping/grasping | LORETA | 0.63 | 61-70 | Algorithm and visual/manual correction |
| 17 | (Divekar and John, 2013) | M/F | Wrist exertions | CMC | 2.34 | 10-20 |  |
| 18 | (Liu et al., 2007) | M | Gripping/grasping | MRCP | 4.5 | 61-70 | Visual/manual correction |
| 19 | ( Liu et al., 2005b) | M | Gripping/grasping | MRCP | 3.22 | 61-70 | Noise source minimization |
| 20 | (Ng and Raveendran, 2007) | M/F | Gripping/grasping | Peak alpha frequency (PAF) | 2.84 | 61-70 | EOG signals correction |
| 21 | (Ng and Raveendran, 2011) | M | Gripping/grasping | Power Spectral Density (PSD) | 0.25 | 61-70 | Blind source separation and wavelet method |
| 22 | (Slobounov et al., 2002) | M | Finger movement | Movement related potential (MRP) | 3.24 | 10-20 | Neuroscan software |
| 23 | (Yang et al., 2009) |  | Gripping/grasping | EEG-EMG coherence | 5.4 | 101-150 | Visual/manual correction |
| 24 | (Amo et al., 2016) |  | Wrist exertions | ERS/ERP | 1.34 | 31-40 | Visual/manual and automatic correction |
| 25 | (Nakayashiki et al., 2014) | M/F | Gripping/grasping | ERD | 3.4 | < 10 |  |
| 26 | (Calmels et al., 2006) |  | Finger movement | Synchronization likelihood | 3.7 | 10-20 |  |
| 27 | (Schwarz et al., 2018) |  | Gripping/grasping | Trial classification, Movement related cortical potential (MRCP) | 10 | 61-70 | Trials rejection, channel rejection |
| 28 | (Vlaar et al., 2018) | M/F | Wrist exertions | Non linear (Volterra series) | 1 | 101-150 | ICA |
| 29 | (Li et al., 2018) |  | Hand and arm movement | Brain Functional Network (BFN), Hierarchical Linear Model (HLM) | 1 | 31-40 | EOG based rejection |
| 30 | (Hübner et al., 2018) | M/F | Gripping/grasping |  | 1 | 31-40 | Bad channel interpolation, ICA and semiautomatic artifact rejection |
| 31 | (Breitling et al., 1986) | F | Finger movement | Band amplitudes spectrum | 0.64 | 10-20 | Visual/manual correction |
| 32 | (Oda et al., 1996) | M | Hand and arm movement | MRCP | 0.66 |  |  |
| 33 | (Shibata et al.,1997) | M | Hand and arm movement | MRCP | 0.91 | <10 |  |
| 34 | (Siemionow et al., 2000) | M | Hand and arm movement | MRCP | 7.16 | <10 |  |
| 35 | (Krause et al., 1983) | M/F | Hand and arm movement | PSD | 0.56 |  |  |
| 36 | (Bates, 1951) |  | Wrist exertions | MRCP | 2.03 |  |  |
| 37 | (Sclocco et al., 2014) | M/F | Hand and arm movement | EEG-fMRI time-frequecy analysis | 1.8 | 21-30 | Time-frequency analysis |
| 38 | (Yang et al., 2016) | M/F | Isotonic wrist flexion | Coherence | 2 | 101-150 | ICA, DIPFIT |

**Table 2**. Studies investigating physical activities with cognition

| Study No. | Authors | Subjects' gender | Physical activity | Data processing method/parameter | Category | Citation No./Years after publication | Number of electrodes | Artifact correction |
| --- | --- | --- | --- | --- | --- | --- | --- | --- |
| 1 | (Smith and Collins, 2004) | M | Mental and physical muscular contractions | CNV | ERP | 4.2 | 31-40 | Eye movement reduction algorithm, trend correction, visual inspection |
| 2 | (Bullock, 2015) | M/F | Exercise | ERP | ERP | 1.75 | 31-40 | Automatic Artifact Removal toolbox to remove ocular artifacts |
| 3 | (Chang et al., 2015) | M | Exercise | ERD | ERP | 5.25 | 21-30 | EOG activity correction |
| 4 | (Chu et al., 2016) |  | Exercise | ERD/ERS | ERP | 0.67 | 31-40 | Eye blink and movement correction with EOG, automatic trial rejection based on a threshold |
| 5 | Magnie et al., 2000) |  | Cycling | ERP | ERP | 6.64 | <10 | Automatic online rejection for P300, offline rejection for N400 |
| 6 | (Wascher et al., 2014) | M/F | Sorting boxes | ERP | ERP | 6 | 21-30 | Automatic rejection of technical artifacts, regression based ocular correction |
| 7 | (Wascher et al., 2016) |  | Sorting boxes | ERD/ERS and power spectral | ERP | 2.34 | 51-60 | Manual rejection of blinks, ICA, ADJUST, DIPFIT |
| 8 | (Themanson and Hillman, 2006) | M/F | Treadmill walking/running | ERP |  | 12.16 | 10-20 | Eyeblink correction with a spatial filter |
| 9 | (Van Cutsem et al., 2017) | M | Cycling | ERP/Power spectral analysis | ERP | 2 | 31-40 | Semiautomatic removal |
| 10 | (De Tommaso et al., 2015) | M/F | Walking | EEG-EMG co-registration | ERP | 3.75 | 21-30 |  |
| 11 | (Killane et al., 2013) | M/F | Exercise | ERP | ERP | 1.84 | <10 | Visual inspection to remove eyeblinks, threshold based rejection on EMG data |
| 12 | (Allami et al., 2014) |  | Gripping/grasping | ERP | ERP | 2.6 | 31-40 | Channel rejection containing muscular or ocular artifacts |
| 13 | (Bullock et al., 2017) | M/F | Cycling | Feature selective profile | Non-ERP | 3.5 | 61-70 | Epochs and trials rejection based on a threshold |
| 14 | (Henz and Schoellhorn, 2017) | M/F | Qigong Training | Power spectral density | Non-ERP | 2 | 10-20 | Visual rejection of blinks, eye, head and muscle movement |
| 15 | (Mierau et al., 2009) | M | Treadmill exercise | Power spectral density | Non-ERP | 2.4 | 10-20 | Visual and automatic artifact detection |
| 16 | (Mikicin and Kowalczyk, 2015) |  | Ergometer swimming | Beta amplitudes | Non-ERP | 0 | 21-30 |  |
| 17 | (Shinohara, 2014) | M/F | Matching task with index finger, Leg resistance exercise | Corticomuscular coherence | Non-ERP | 0 |  |  |
| 18 | (De Hillerin et al., 2015) |  | Exercise | EEG asymmetry | Non-ERP | 0 | 10-20 |  |
| 19 | (Kim et al., 2018) | M/F | Finger tapping | Dynamic Casual Modeling (DCM) | Non-ERP | 6 | 31-40 | ICA, Lowest Amplitude based artifact rejection, interpolation of bad channels based on eye and muscular movement |
| 20 | (Bigliassi et al., 2018) | M/F | Isometric ankle-dorsiflexion | Source reconstruction with full-frequency spectrum analysis | Non-ERP | 5 | 61-70 | ICA and visual inspection |
| 21 | (Blinowska et al., 2008) |  | Finger movement | Short-time Directed Transfer Function (SDTF) | Non-ERP | 0.28 | 21-30 | Removal of 50 Hz artifacts |
| 22 | (Pires et al., 2018) | M | Cycling | Alpha wave at primary motor cortex | Non-ERP | 2 |  |  |

**Table 3**. Studies investigating miscellaneous physical activities

| Study No. | Authors | Subjects' gender | Physical activity | Data processing method/parameter | Category | Citation No./Years after publication | Number of electrodes | Artifact correction |
| --- | --- | --- | --- | --- | --- | --- | --- | --- |
| 1 | (Barlaam et al., 2011) | M/F | Bimanual load lifting | Time-frequency analysis, ERP | Neural correlates without mental effects | 1.13 | 61-70 | MATLAB and BrainVision software |
| 2 | (Bruijn et al., 2015) | M/F | Walking | Spectral analysis | Neural correlates without mental effects | 2 | 61-70 | Visual/manual correction and channel rejection |
| 3 | (Castermans et al.,2014) | M/F | Walking | Time-frequency analysis | Neural correlates without mental effects | 10.8 | 31-40 | Trend correction/rejection |
| 4 | (Presacco et al.,2011) | M/F | Walking | Power Spectral Density | Neural correlates without mental effects | 14.88 | 10-20 | Frontal/temporal electrodes rejection |
| 5 | (Gwin et al.,2011) |  | Walking | Event related spectral perturbation (ERSP) | Neural correlates without mental effects | 25.38 | 201-250 | AMICA and channel rejection |
| 6 | (Gallicchio et al., 2016) | M/F | Biathlon shooting | Power Spectral Density | Neural correlates with mental effects | 1.67 | 61-70 | Visual/manual correction and ICA |
| 7 | (Lattari et al., 2016) |  | Exercise | Power Spectral Density | Neural correlates with mental effects | 0.34 | 10-20 | Visual/manual correction and ICA |
| 8 | (Wieser et al., 2010) | M/F | Walking/gait | sLORETA | Neural correlates without mental effects | 6 | 61-70 |  |
| 9 | (Seeber et al., 2014) | M/F | Walking/gait | Time-frequency decomposition | Neural correlates without mental effects | 10.6 | 101-150 |  |
| 10 | (Storzer et al., 2016) |  | Cycling and Walking | Power Spectral Analysis | Neural correlates with other recording methods | 2.34 | 10-20 | Visual/manual correction and ICA |
| 11 | (Ftaiti et al., 2010) |  | Cycling | Alpha-beta ratio | Neural correlates without mental effects | 3.67 |  |  |
| 12 | (Nybo and Nielsen, 2001) |  | Cycling | Power spectrum, alpha-beta ratio | Neural correlates with mental effects | 14.73 |  |  |
| 13 | (Tuncel et al., 2010) | M | Lifting | EEG-EMG coherence | Neural correlates with mental effects | 2.45 |  | Filters only |
| 14 | (Zhang et al., 2011) |  | Exercise | EEG alpha index and EMG parameters | Neural correlates with other recording methods | 0.25 | <10 |  |
| 15 | (Bigliassi et al., 2017) | M/F | Cycling | Time frequency and spectral coherence | Neural correlates with mental effects | 3 | 31-40 |  |
| 16 | (Hall et al., 2010) | M/F | Treadmill walking/running | EEG asymmetry, power spectral density | Neural correlates with mental effects | 3.12 | 10-20 | Visual/manual correction and EOG signal correction |
| 17 | (Hicks et al., 2018) | M/F | Aerobic exercise and bilateral movement | Power spectral density | Neural correlates with mental effects | 0 | 31-40 | Visual/manual correction and ICA |
| 18 | (Ludyga et al., 2016) | M/F | Cycling | Power Spectral Density | Neural correlates without mental effects | 3.34 | 31-40 | Systematic protocol |
| 19 | (Mizelle et al., 2011) | M/F | Tool handling | sLORETA | Neural correlates without mental effects | 1.63 | 51-60 | Visual/manual correction |
| 20 | (Moraes et al., 2011) |  | Cycling | sLORETA | Neural correlates with mental effects | 4.25 | 10-20 | Visual/manual correction, ICA and epochs rejection |
| 21 | (Schneider et al., 2009a) | M/F | Cycling | Power Spectral Analysis | Neural correlates with mental effects | 2.8 | <10 |  |
| 22 | (Schneider et al., 2009b) | M/F | Treadmill exercise | Power Spectral Analysis | Neural correlates with mental effects | 8.2 | 10-20 | Standard threshold method |
| 23 | ( Schneider et al., 2009c) | M/F | Arm crank–bike–treadmill | sLORETA | Neural correlates with mental effects | 6.3 | 61-70 | Visual/manual correction and semiautomatic ICA |
| 24 | (Woo et al., 2010) | F | Treadmill walking/running | Frontal alpha asymmetry | Neural correlates with mental effects | 2.23 |  | Visual/manual correction |
| 25 | (Comstock et al., 2011) | M | Squats | motor related cortical potential activity (MRCP) | Neural correlates without mental effects | 0 | 31-40 | Eye electrodes signal correction and spatial filter |
| 26 | (Zadry et al., 2010) | M/F | Assembling and disassembling weights | EEG alpha power | Neural correlates without mental effects | 0 |  |  |
| 27 | (Choktanomsup et al., 2017) | M | Moderate intensity running | Power Spectral Analysis | Neural correlates without mental effects | 0 |  |  |
| 28 | (Engchuan et al., 2017) |  | Weight training exercise | Power Spectral Analysis | Neural correlates without mental effects | 0 |  |  |
| 29 | (Winslow et al,, 2016) | M | Walking | Corticomuscular Coherence | Neural correlates with other recording methods | 1.34 | 61-70 | Visual/manual correction and ICA |
| 30 | (Hilty et al., 2011) | M | Cycling | Phase synchronization, sLORETA | Neural correlates with mental effects | 7.25 | 101-150 | ICA |
| 31 | (Bailey et al., 2008) | M | Cycling | Power spectra | Neural correlates without mental effects | 5.64 | 21-30 | Visual/manual correction |
| 32 | (Rasmussen et al., 2004) | M | Cycling | Power spectra | Neural correlates with mental effects | 3.34 |  |  |
| 33 | (Dal Maso et al., 2012) |  | Strength training | Power spectra | Neural correlates with other recording methods | 3.58 | 61-70 | ICA |
| 34 | (Flanagan et al., 2012) | M | Squats | EEG potentials | Neural correlates with mental effects | 0.72 | 31-40 | Eye electrodes signal correction |
| 35 | (Spring et al., 2017) | M | Cycling | Power spectra, Microstate analysis | Neural correlates without mental effects | 0 | 61-70 | Visual/manual correction and ICA |
| 36 | (Dal Maso et al., 2018) |  | Cycling and gripping | ERD | Neural correlates with other recording methods | 6 | 61-70 | Visual/manual correction and ICA |
| 37 | (Daou et al., 2018) | M/F | Golf putting | Power spectral density | Neural correlates without mental effects | 3 | 61-70 | Visual/manual correction and ICA |
| 38 | (Périard et al., 2018) | M | Cycling | sLORETA | Neural correlates with mental effects | 3 | 61-70 | Visual/manual correction, ICA and inverse ICA |
| 39 | (Peterson and Ferris, 2018) | M/F | Walking, standing | PSD, ERSP | Neural correlates without mental effects | 1 | 101-150 | ASR, ICA, PCA, DIPFIT |
| 40 | (Salazar et al., 1990) | M/F | Archery | PSD | Neural correlates without mental effects | 6.66 |  | EOG signal correction |
| 41 | (Kubitz and Mott, 1996) | M/F | Cycling | PSD | Neural correlates without mental effects | 3.96 |  |  |
| 42 | (Collins et al., 1990) | M | Breaking a wooden board | Band power (PSD) | Neural correlates without mental effects | 1.8 |  |  |
| 43 | (Kubitz and Pothakos, 1997) | M/F | Cycling | PSD | Neural correlates without mental effects | 2.96 |  | Visual/manual correction |
| 44 | (Beyer et al., 1989) |  | Cycling | PSD | Neural correlates without mental effects | 0 |  |  |
| 45 | (Daniels et al., 1984) |  | Running and cycling |  | Neural correlates without mental effects | 0 |  |  |
| 46 | (Kakizaki, 1988) | M | Cycling | Oz Average amplitudes | Neural correlates without mental effects | 0 |  |  |
| 47 | (Dostalek et al., 1980) |  | Yoga exercise | BPSW (Biphasic Paroxysmal Sharp Waves) | Neural correlates without mental effects | 0 |  |  |
| 48 | (Gotze et al., 1966) |  | Knee bending exercise |  | Neural correlates without mental effects | 0.02 |  |  |
| 49 | (Wales and Thayer, 1985) | M | Cycling | Temporal beta activity | Neural correlates with mental effects |  |  |  |
| 50 | (Lin et al., 2017) | M/F | Exercise | Spectral power, Fuzzy entropy | Neural correlates with other recording methods | 0 | <10 | Visual/manual correction |
